# Prostate Abscess Caused by Community-Acquired Methicillin-Resistant *Staphylococcus aureus*

**DOI:** 10.1177/2324709618788899

**Published:** 2018-07-18

**Authors:** Asad Ullah, Zeeshan Khakwani, Hassan Mehmood

**Affiliations:** 1 Conemaugh Memorial Medical Center, Johnstown, PA, USA

**Keywords:** MRSA, methicillin-resistant *Staphylococcus aureus*, prostate abscess

## Abstract

A 60-year-old male presented with complaints of fever, chills, cough, and shortness of breath. He denied abdominal pain, urgency, dysuria, or hematuria. His laboratory data revealed an elevated white blood cell count and lactic acid, and one set of blood culture stained positive for gram-positive cocci. He was empirically started on intravenous antibiotics. On the next hospital day, the patient complained of hematuria and lower abdominal discomfort. A computed tomography scan of his abdomen and pelvis was obtained that revealed a focal hypodensity relating to prostate abscess, which was subsequently confirmed on magnetic resonance imaging of the prostate. Final report of blood cultures stated methicillin-resistant *Staphylococcus aureus*. He was treated with intravenous vancomycin and transurethral resection and deroofing of the prostate gland with drainage that resulted in complete resolution of his symptoms.

## Introduction

Prostate abscess is a rare disease due to widespread use of antibiotics for lower urinary tract infections.^1,2^ It can be a life-threatening disorder and had a mortality rate of 6% to 30% before the advent of effective antibiotics therapy.^3^ Most cases present with nonspecific signs and symptoms, which makes an early diagnosis challenging.^4^ It is most commonly caused by *Escherichia coli* and other gram-negative bacteria. *Staphylococcus aureus* has been increasingly isolated as the causative bacteria, especially methicillin-resistant strains, in patients with diabetes and urinary tract abnormalities.^5^ Magnetic resonance imaging (MRI) and transrectal ultrasonography (TRUS) are very helpful in diagnostic and therapeutic processes. In the past, prostate abscess was surgically managed by perineal or transrectal incision but recently less invasive surgical procedures like TRUS-guided needle aspiration is preferred as it is associated with fewer complications and a better outcome.^5^

## Case

A 60-year-old male presented to our emergency department with complaints of sudden onset of fever, chills, shortness of breath, and nonproductive cough for 1 day. He also reported having malaise, nausea, and rapid heartbeat. His past medical history included type 2 diabetes mellitus, hypertension, benign prostatic hyperplasia, deep venous thrombosis, hyperlipidemia, and chronic kidney disease stage 3. He denied any associated abdominal pain, diarrhea, dysuria, frequency, hematuria, or perineal discomfort.

In the emergency department, his temperature was 40.1°C, blood pressure was 111/51 mm Hg, heart rate was 121 beats per minute, and oxygen saturation was 95% on room air. He was alert, awake, and oriented. Pulmonary, cardiovascular, abdominal, and neurological examinations were unremarkable.

Laboratory data were significant for white blood cell count of 11.4 × 10^3^/uL (reference: 4.5-11 × 10^3^/µL), serum creatinine of 1.3 (reference: 0.7-1.3 mg/dL), lactic acid of 3.0 mg/dL (reference: 0.5-2.2 mg/dL), and glucose of 355 mg/dL (reference: 80-115 mg/dL). Urinalysis showed 10 to 15 red blood cells/high-power field, with other parameters within normal limits. Liver function tests were normal. Chest X-ray showed chronic hyperventilatory changes. Contrast-enhanced computed tomography (CT) scan of lungs did not reveal any pulmonary embolism or obvious lung consolidation. One set of blood cultures obtained grew gram-positive cocci. Urine culture was pending.

**Figure 1.**
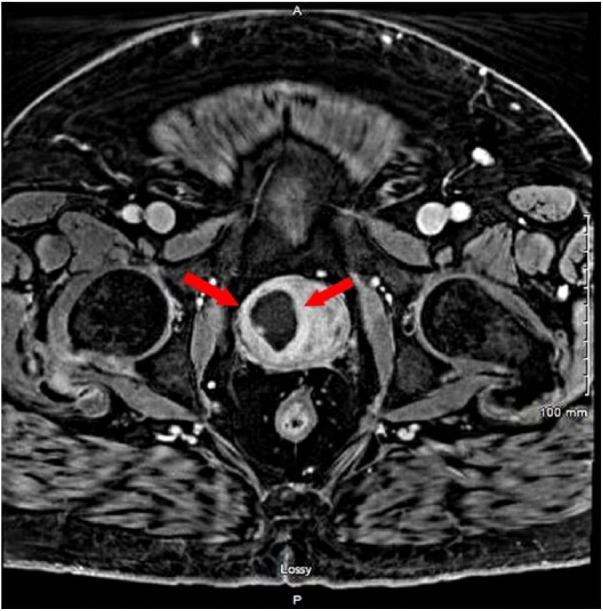
Magnetic resonance imaging axial view showing cystic lesion (red arrows) within the right aspect of prostate gland with marked restriction of diffusion.

**Figure 2.**
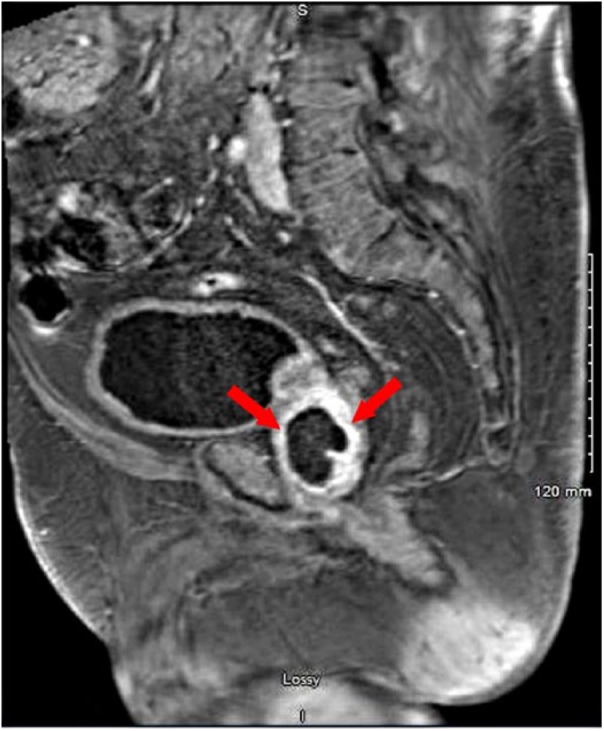
Magnetic resonance imaging sagittal view showing rim enhancing prostatic lesion (red arrows).

The patient was empirically started on intravenous (IV) vancomycin. He continued to have fever and chills, and also reported lower abdominal discomfort and hematuria. As there was no exact source of bacteremia at this point, CT scan of his abdomen and pelvis was obtained for further evaluation, which revealed a focal hypodensity in the right side of the prostate that could be related to a neoplasm or an abscess. Urology team was consulted and a second set of blood cultures was obtained, and IV piperacillin-tazobactam was added to his antibiotic regimen for gram-negative coverage. Subsequently, MRI of prostate confirmed the findings of CT scan, revealing an enlarged prostate pressing on the base of the urinary bladder and a cystic lesion measuring 3.7 × 2.5 × 3.8 cm within the right aspect of the prostate gland (Figures 1 and 2). Final result of first and second sets of blood culture reported methicillin-resistant *Staphylococcus aureus* (MRSA). Subsequently, a third set of blood culture was also positive for MRSA, making it a total of 3 sets of blood cultures positive for MRSA.

The patient underwent transurethral resection and unroofing of the prostate abscess with drainage, and a Foley catheter was placed for urinary drainage. He had significant improvement of his symptoms. Foley catheter was taken out, and the patient was able to void without any problems. Tissue pathology reported benign prostatic chips exhibiting foci of necrosis with mixed inflammation and scattered bacterial colonies, features consistent with abscess. Repeat sets of blood and urine cultures did not report any growth, showing adequate response to treatment. A peripherally inserted central line was placed as the patient required IV vancomycin therapy, and he was discharged in a stable condition. Patient was seen at our hospital by our Urology team about 3 weeks later, and he did not have any urologic issues. Blood and urine cultures were also repeated that did not report growth of any organism. He received a total of 1 month of antibiotic therapy.

## Discussion

S*taphylococcus* aureus, a gram-positive bacteria, is a prominent human pathogen that can cause a diverse range of diseases ranging from relatively minor skin infections to serious and life-threatening infections such as endocarditis, pneumonia, and sepsis.^6^ Its impact is enhanced by the development of antibiotic resistance, most notably MRSA.^6^ It has become a major public health problem primarily related to health care but no longer confined to health care institutions^7^ but also with increased prevalence in the community.^8^ Recently, there has been a significant increase in the incidence of skin and soft tissue infections caused by community-acquired MRSA (CA-MRSA), which now accounts for most infections caused by *S aureus*.^8^

Prostate abscess is a rare infection of the prostate gland due to widespread use of broad-spectrum antibiotics for lower urinary tract infections.^1,2^ It has a wide spectrum of clinical presentation that overlaps with other urinary tract infections, which makes the diagnosis difficult.^4^ It can result in significant morbidity and even mortality, and thus requires prompt diagnosis and treatment.^9^ In the pre-antibiotic era, *Neisseria gonorrhea* was the most common cause of prostate abscess^10^; now *E coli* and other gram-negative bacteria are responsible for the majority of the cases.^11^ Recently, *S aureus* has been increasingly isolated as the culprit organism causing prostate abscess, a literature review identifying 39 cases of which 26 were methicillin resistant.^12^

CA-MRSA is a rare cause of prostate abscess and is mostly identified in patients with diabetes mellitus, immunocompromised states, chronic hemodialysis, chronic indwelling catheterization, and instrumentation of the lower genitourinary tract.^10^

There are different factors that have been described in medical literature that can predispose an individual for developing a prostate abscess, which includes retrograde flow of contaminated urine, complication of acute or chronic prostatitis, or hematogenous spread of infection from a distant locus. Patients usually present with fever, dysuria, frequency, urgency, urinary retention, and perineal pain.^1^ The typical finding on physical examination is a fluctuating area in the prostate gland, while pain on palpation on digital rectal examination is universal among patients with the disease.^1^

There is no standardized diagnostic and therapeutic approach in managing patients with prostate abscess. Imaging modalities including CT, MRI, and TRUS play a vital role in diagnosis.^10^ CT and MRI are useful to assess the extent of suppurative collection in the periprostatic tissue and to detect gas in the fluid.^10^ But the diagnostic study of choice is TRUS that can aid in localizing the abscess and also help in needle aspiration and following response to surgical therapy.^13^

Treatment of prostate abscess includes administration of parental antibiotics and surgical drainage.^13^ Broad-spectrum antibiotics with adequate coverage for *E coli* and MRSA should be administered, which can be tailored according to culture and sensitivity reports of blood, urine, or tissue samples. Surgical procedures like perineal incision and transurethral resection were the first-line therapies for the surgical management of prostate abscess.^14^ More recently, TRUS-guided needle aspiration transrectally or transperineally is preferred as it is minimally invasive and has less complications with a significantly better outcome.^15,16^

## Conclusion

CA-MRSA is an emerging cause of prostate abscess, mostly affecting individuals with diabetes and urinary tract abnormalities. Transrectal ultrasonography and MRI are valuable imaging modalities in diagnosing prostate abscess as well as treatment. Broad-spectrum antibiotics coupled with surgical procedures like transurethral resection or TRUS-guided needle aspiration are the mainstays of the treatment resulting in complete remission in majority of patients.
